# The prognostic value of abnormally expressed lncRNAs in colorectal cancer: A meta-analysis

**DOI:** 10.1371/journal.pone.0179670

**Published:** 2017-06-28

**Authors:** June Wang, Shenlin Du, Jiamin Wang, Wei Fan, Ping Wang, Zheng Zhang, Peipei Xu, Shihui Tang, Qiaoling Deng, Weiqing Yang, Mingxia Yu

**Affiliations:** 1 Department of Clinical Laboratory & Center for gene diagnosis, Zhongnan Hospital of Wuhan University, Wuhan, China; 2 Guangdong Provincial Key Laboratory of Medical Molecular Diagnostics, Guangdong Medical University, Dongguan, China; 3 Department of Pathology, Zhongnan Hospital of Wuhan University, Wuhan, Hubei, China; University of South Alabama Mitchell Cancer Institute, UNITED STATES

## Abstract

**Background:**

Colorectal cancer (CRC) is the third most prevalent cancer type and the third leading cause of cancer-related deaths worldwide, it is urgently needed to discover a new marker for the progress of CRC. Many long noncoding RNAs (lncRNAs) have been reported to be abnormally expressed in CRC, and may be feasible as effective biomarkers and prognostic factors. The aim of this study was to identify the prognostic value of various lncRNAs in CRC.

**Methods:**

Pubmed, Web of Science, Embase and Cochrane Library were searched for potentially related studies. A total of 34 eligible studies including 30 on overall survival (OS), 7 on disease-free survival (DFS), 1 on relapse-free survival (RFS), 2 on disease-specific survival (DSS) and 29 on clinicopathological features were qualified from the databases.

**Results:**

The results showed that the expression levels of lncRNAs were significantly associated with poor OS (hazard ratio (HR) = 2.08, 95% confidence interval (CI) = 1.68–2.57, *P*<0.001, *I*^2^ = 70%), DFS (HR = 1.79, 95% CI = 1.54–2.08, *P*<0.001, *I*^2^ = 6%) and DSS (HR = 0.11, 95% CI = 0.02–0.54, *P* = 0.007, *I*^*2*^ = 14%). Subgroup analysis further showed that lncRNA transcription level was significantly associated with tumor differentiation (odds ratio (OR) = 0.51, 95% CI = 0.34–0.77, *P* = 0.001), lymph node metastasis (OR = 1.63, 95% CI = 1.23–2.17, *P* = 0.0007), distant metastasis (OR = 2.06, 95% CI = 1.29–3.30, *P* = 0.002), TNM stage (OR = 0.44, 95% CI = 0.32–0.62, *P*<0.001), tumor invasion depth (OR = 0.48, 95% CI = 0.39–0.60, *P*<0.001).

**Conclusions:**

The meta-analysis demonstrated that abnormal lncRNA transcription level may serve as a promising indicator for prognostic of patients with CRC.

## Introduction

Colorectal cancer (CRC) is one of the most common malignancies, which ranks the third in the cancer morbidity and the second in the cancer mortality worldwide, with an annual 1.3 million new CRC cancer cases and 694,000 deaths according to the GLOBOCAN estimations[1, 2]. Although advancements have been made regarding the available treatment strategies, the overall survival rate of CRC patients has not improved dramatically[3, 4]. Relapse and metastasis are major factors for the poor outcome of CRC patients[5]. The occurrence of CRC involves multi-factorial and complex steps in which the abnormal gene expression plays a vital role[6]. Therefore, it is emergently necessary to identify novel molecular markers for the early detection, prognosis prediction and therapy evaluation for CRC.

Long non-coding RNAs (lncRNAs), which are defined as RNA molecules of larger than 200 nucleotides in length without protein-coding capacity, regulate gene expression at the epigenetic, transcriptional or posttranscriptional level and once were considered to be transcriptional noise[7–9]. Recently, accumulating evidences showed that lncRNAs played pivotal roles in various cancers and were associated with tumor cell proliferation, apoptosis, invasion and metastasis[10, 11]. For example, plasmacytoma variant translocation 1 gene (PVT1) could inhibit the apoptosis of breast cancer cells [12]. LncRNA H19 was significantly up-regulated in the plasma of Gastric Cancer (GC) patients, and could be a potential non-invasive diagnostic biomarker in GC[13]. In 2016 Huang *et al*. [14] reported that lncRNA DGCR5 was down-regulated in hepatocellular carcinoma and correlated with poor prognosis. In non-small cell lung cancer, upregulation of long non-coding RNA ATB, a TGF-β-activated lncRNA, indicated a poor prognosis by regulating cell proliferation and metastasis[15]. Recent mounting studies have shown that lncRNAs are potential diagnostic and prognostic biomarkers of CRC.

Up to now, some lncRNAs have been shown to be expressed aberrantly in CRC, such as SNHG20[16], TUG1[17], and 91H[18]. In 2015, Chen *et al*. [19] found that FEZF1 antisense RNA1 (FEZF1-AS1) was upregulated in CRC tissues and could serve as a potential therapeutic target in CRC. Colon cancer-associated transcript 1 (CCAT1) activated by c-Myc, plays an oncogenic role in CRC development and metastasis[20]. Promoter of CDKN 1A antisense DNA damage activated RNA (PANDAR) could affect epithelial–mesenchymal transition through inhibiting N-cadherin, vimentin, β-catenin, Snail and Twist expression and increasing the expression levels of E-cadherin. The results indicted that PANDAR could be a biomarker for poor prognosis of CRC[21]. Many studies were performed to investigate the prognostic value of lncRNAs in CRC. However, single study may be inaccurate and insufficient, Thus, studies should be analyzed systematically to gain a better insight into the potential clinical values of lncRNAs in CRC. Although some reviews have reported evaluation of the clinical values of multiple lncRNAs in CRC, meta-analysis of lncRNAs in CRC has not yet to be performed. Therefore, relevant articles were collected to evaluate the relationship between lncRNAs expression and clinical outcomes in CRC.

## Materials and methods

### Publication search

We retrieved Pubmed, Web of Science, Embase and Cochrane Library to obtain all relevant articles. The literature search was limited to the English language and ended in January 22, 2017. The search strategy used both MeSH terms and free-text words to increase the sensitivity of the search. The search terms included: (“Long non-coding RNA”, “lncRNA”, “LincRNA”, “Long ncRNA”, “Long intergenic non-coding RNA”) AND (“CRC”, “colorectal cancer”, “colorectal neoplasm”, “colorectal tumor”, “rectal neoplasm”, “rectal cancer”, “rectal tumor”, “colon neoplasm”, “colon cancer”, “colon tumor”). Relevant articles were also reviewed manually in case of the omission of any potentially relevant literature.

### Inclusion and exclusion criteria

The eligible studies met the following criteria: patients were diagnosed with colorectal cancer; relationship between lncRNAs and colorectal cancer was investigated; the prognostic value of lncRNAs was evaluated; the association between lncRNA expression and survival (OS, disease-free survival [DFS], and disease-specific survival [DSS]) was performed; and the survival curve or sufficient relevant data was provided to obtain hazard ratios (HR) for survival rates and their 95% confidence intervals (95% CI). Exclusion criteria were as follows: duplicate studies; sample population consisted of less than 40 cases; non-English papers; letters; review articles; case reports; lack of original data; non-human studies.

### Data quality assessment and extraction

Two investigators (JEW and SLD) extracted and reviewed the essential information of each eligible study independently according to pre-specified inclusion and exclusion criteria. The following data were extracted: the first author’s name, year of publication, country, sample size, tumor type, cutoff value, detection method, outcome, analysis type and quality score. The quality of the included studies was assessed with the Newcastle-Ottawa Scale (NOS) criteria for cohort studies [22]. HRs and their 95% CIs were extracted directly from the original articles or calculated from Kaplan-Meier survival curve by HR digitizer software Engauge 4.1 as described by Thierny et al[23]. Any discrepancies on data extraction and quality assessment were resolved through discussion with a third reviewer (MXY). The quality of all the included studies was assessed by The Newcastle-Ottawa Scale (NOS) method. The NOS scores ranged from 0 to 9, and a study with the NOS score more than 6 was regarded as high quality.

### Statistical analysis

Statistical analysis was conducted with Review Manager5.2 (The Cochrane Collaboration, Software Update, Oxford, UK) and stata12.0 (STATA Corporation, College Station, TX, USA). HRs and its 95% CI were used to evaluate the association between lncRNAs and survival in CRC. HR > 1 indicated that the patients with high lncRNAs expression had a poor prognosis. Conversely, HR<1 implied the patients with low lncRNAs expression had a good prognosis. ORs and 95% CIs were used to assess the association between lncRNAs and clinical features in patients of CRC. The features included gender, tumor size, tumor differentiation, distant metastasis, lymph node status, TNM stage and tumor invasion depth. Heterogeneity among the eligible studies was assessed with the Q test and *I*^2^ statistic, and the *I*^2^ value indicated the degree of heterogeneity. A *p*-value<0.05 or *I*^2^>50% indicated significant heterogeneity, in which case a random-effects model was used, if not, a fixed-effects model was used. Publication bias was evaluated by Begg’s test and *P*>0.05 indicated no significant bias among studies. Sensitivity analyses were performed to access the stability of the meta-analysis results. All the *P* values were determined by two-sided tests.

## Results

### Study characteristics

**Fig 1:**
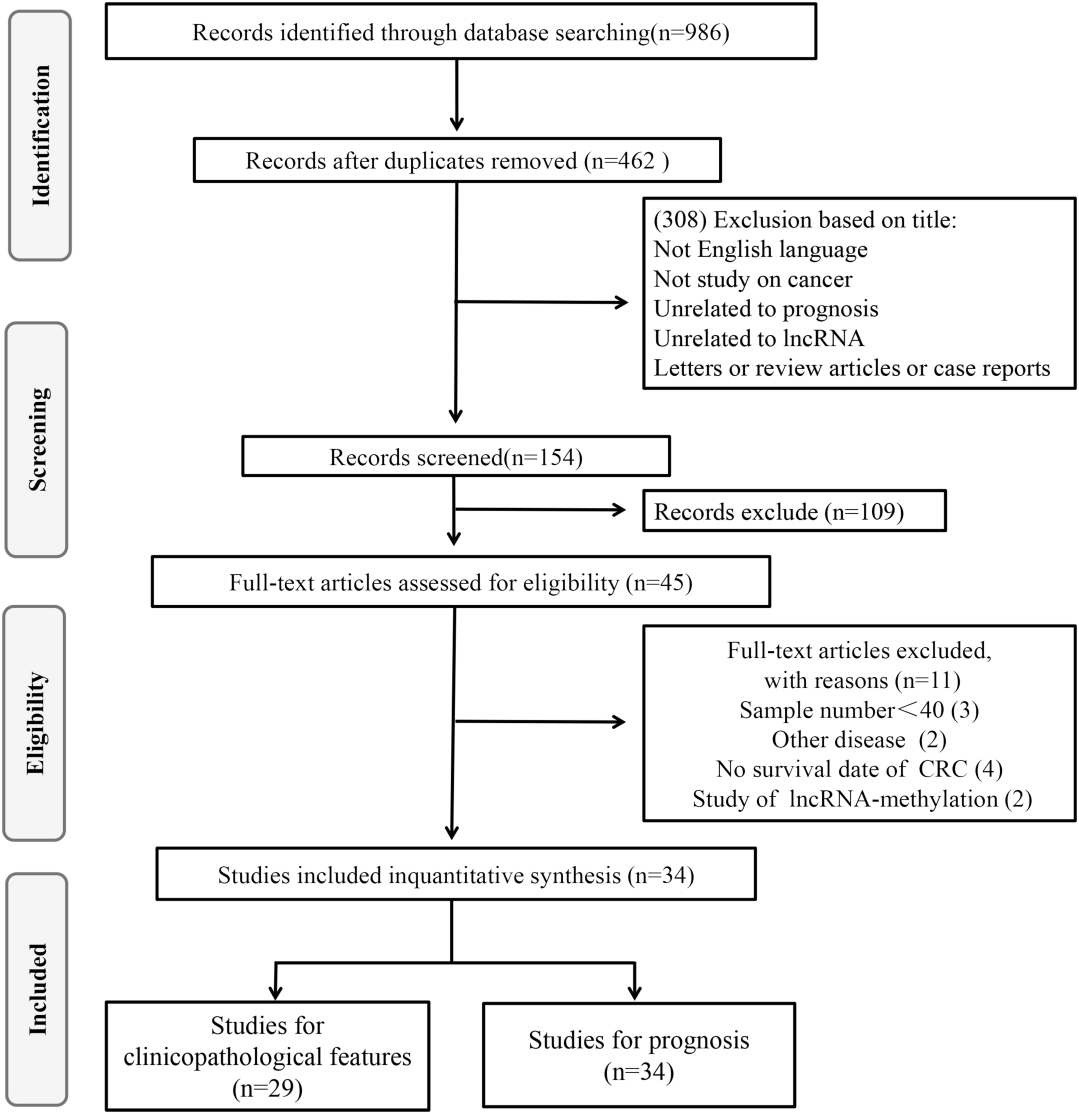
The flow diagram of this meta-analysis in CRC.

As shown in the flow diagram (Fig 1), a total of 986 records were retrieved from Pubmed, Web of Science, Embase and Cochrane Library. 524 duplicate reports were excluded. After the titles and abstracts were reviewed, 308 irrelevant articles were excluded. Subsequently, the 154 remaining full-text articles were assessed, 120 were found to be ineligible due to lack of sufficient data for further analysis, 34 studies were eligible for the current meta-analysis, including 30 on OS[16–20, 24–48], 7 on DFS[19, 25, 34, 36, 37, 42, 49], 1 on RFS[24], 2 on DSS[50, 51], and 29 on clinicopathological features[16–20, 24–29, 31, 32, 34–37, 39–46, 48–51].

Among these 34 studies, a total of 3653 patients were represented. The mean size of patient sample was 111 (range from 48 to 239). The most recent publication date was January 22, 2017. Among the eligible articles, 31 were from China, 3 from Japan and 1 from Czech Republic. In the including 34 studies, 29 articles analyzed the relationship between the expression of lncRNAs and gender[16–20, 24–29, 31, 32, 34–37, 39–46, 48–51], 7 studies estimated the association between lncRNAs and tumor size[19, 24, 25, 27, 41, 42, 48], 16 studies estimated the association between lncRNAs and tumor differentiation[16, 17, 19, 24, 25, 28, 29, 34, 36, 37, 39, 40, 43, 45, 46, 48], 24 studies were about lymph node metastasis(LNM)[16–20, 26, 27, 29–32, 34–37, 39, 40, 42–46, 48, 50], and 20 were about distant metastasis[16, 18–20, 25, 27–29, 35–37, 39, 40, 42–46, 48, 50], 18 studies demonstrated that lncRNAs were correlated with TNM stage[18, 20, 25, 27, 29, 35–39, 41, 42, 44, 46, 48, 50–52], 17 studies reported the association between lncRNAs and tumor invasion depth[16, 18, 19, 26–28, 31, 36, 39, 41, 42, 44–46, 48, 50, 51]. The main information and data were summarized in Table 1.

**Table 1:** Characteristics of studies included in this meta-analysis.

Author Year of publication	Country	LncRNAs	Sample size (high/low)	Tumor type	Cutoff	Detection method	Outcome	Analysis type	Quality score
Qi2013	China	LOC285194	33/48	CRC	mean	qRT-PCR	DSS	Multivariate	8
Shi2014	China	RP11-462C24.1	32/54	CRC	mean	qRT-PCR	DSS	Multivariate	8
Li2016	China	SNHG20	54/53	CRC	2.86-fold	qRT-PCR	OS	Multivariate	7
SunJ2016	China	TUG1	38/23	CRC	fivefold	qRT-PCR	OS	Kaplan-Meiercurves	7
SunY2016	China	ANRIL	53/44	CRC	RE1.5	RT-qPCR	OS	Kaplan-Meiercurves	7
Lu2016	China	PANDAR	62/62	CRC	median	qRT-PCR	OS	Multivariate	8
Ge2013	China	PCAT-1	50/58	CRC	ROC	qRT-PCR	OS	Multivariate	7
Yin2014	China	GAS5	33/33	CRC	mean	qRT-PCR	OS	Multivariate	6
Yin2015	China	MEG3	31/31	CRC	mean	qRT-PCR	OS	Multivariate	8
Ye2015	China	CLMAT3	45/45	CRC	RE	qRT-PCR	OS	Multivariate	8
Takahashi2013	Japan	PVT-1	131/33	CRC	MYC expression	qRT–PCR	OS	Multivariate	7
Svoboda2014	Czech Republic	HOTAIR	36/37	CRC	ROC	RT-qPCR	OS	Multivariate	8
WangW2016	China	ZFAS1	79/80	CRC	median	qRT-PCR	RFS/OS	Multivariate	7
WangF2016	China	AFAP1-AS1	26/26	CRC	median	qRT-PCR	DFS/OS	Multivariate	8
Zheng2014	China	MALAT1	73/73	CRC	RE 6.15	qRT–PCR	DFS/OS	Multivariate	7
Iguchi2015	Japan	lncRNA-ATB	62/62	CRC	median	RT-PCR	DFS	Kaplan-Meiercurves	8
Ni2015	China	UCA1	27/27	CRC	median	RT-qPCR	OS	Kaplan-Meiercurves	8
LiY2015	China	NEAT1	110/129	CRC	2-fold	RT-PCR	DFS/OS	Multivariate	8
Han2016	China	H19	48/35	CRC	3-fold	qRT-PCR	DFS/OS	Multivariate	7
Liu2016	China	CRNDE-h	71/71	CRC	median	qRT-PCR	OS	Multivariate	7
Deng2014	China	91H	30/42	CRC	RE2.86	qRT-PCR	OS	Multivariate	6
Wu2014	China	HOTAIR	40/80	CC	5-fold	qRT-PCR	OS	Multivariate	8
Kogo2011	Japan	HOTAIR	20/80	CRC	RE 0.273	qRT-PCR	OS	Kaplan-Meiercurves	7
Han2014	China	UCA1	37/43	CRC	mean	RT-qPCR	OS	Kaplan-Meiercurves	7
He2014	China	CCAT1	24/24	CC	median	qPCR	OS	Kaplan-Meiercurves	8
Liu2015	China	DANCR	52/52	CRC	median	qRT-PCR	DFS/OS	Multivariate	8
Guo2015	China	FTX	75/112	CRC	median	qRT-PCR	OS	Multivariate	7
Ren2015	China	HOTTIP	77/79	CRC	median	qRT-PCR	OS	Multivariate	6
Chen2016	China	FEZF1-AS1	89/64	CRC	-	RT-PCR	OS	Multivariate	7
Chen2016	China	FEZF1-AS1	89/64	CRC	-	RT-PCR	DFS	Kaplan-Meiercurves	8
Cao2016	China	SPRY4-IT1	36/48	CRC	2.87-fold	qRT-PCR	OS	Multivariate	8
Jiang2016	China	UCA1	61/60	CRC	median	qRT-PCR	OS	Multivariate	7
Bian2015	China	UCA1	45/45	CRC	2-fold	qRT-PCR	OS	Multivariate	7
Qiu2015	China	LINC01296	80/80	CRC	GAPDH	GEO	OS	Multivariate	6

Abbreviations: LncRNA: Long-coding RNA; CRC: Colorectal Cancer; CC: Colon Cancer; RE: Relative expression; RT-PCR: reverse transcription -polymerase chain reaction; qPCR: Real-time-PCR; qRT-PCR: Quantities reverse transcription-PCR; OS: Overall survival; DFS: Disease-free survival; DSS: Disease-specific survival; NA:Not available.

### Global analysis between lncRNA transcription level and CRC survival

**Fig 2:**
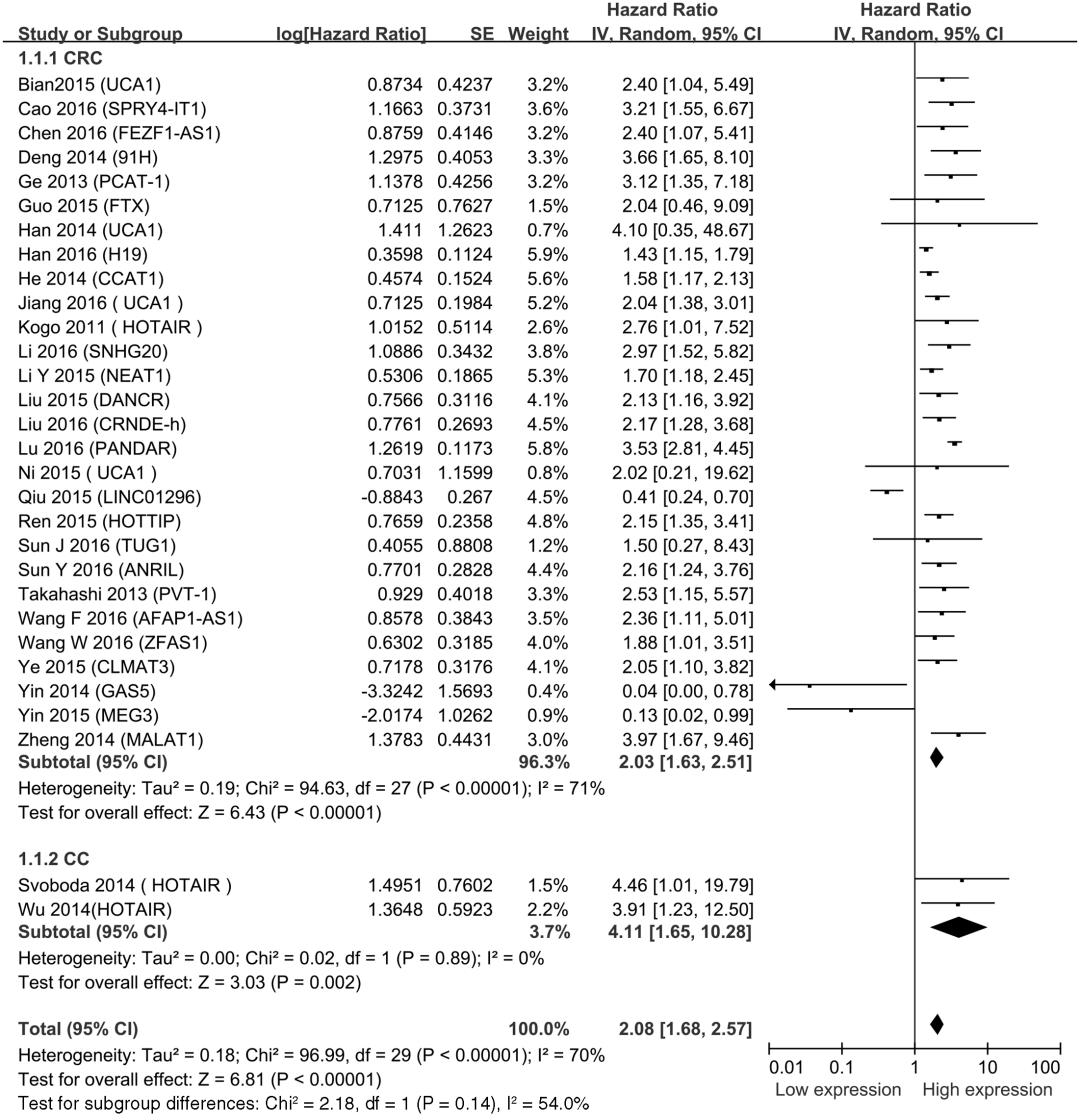
Forest plot for the association between lncRNAs expression levels with overall survival in CRC.

A total of 30 studies of 3361 patients reported OS of CRC. HRs and corresponding 95% CIs of OS were extracted from the included studies. The estimated pooled HR showed a significant association between lncRNAs and OS in CRC patients (HR = 2.08, 95% CI = 1.68–2.57, *P*<0.001, *I*^2^ = 70%) (Fig 2). There are two articles [20, 39] that reported the relationship between the expression level of lncRNAs and OS of colon cancer (HR = 4.11, 95% CI = 1.65–10.28, *P* = 0.002, *I*^2^ = 0%). The increased expressions of SPRY4-IT1, FEZF1-AS1, 91H, PCAT-1, H19, CCAT1, UCA1, HOTAIR, SNHG20, NEAT1, DANCR, CRNDE-h, PANDAR, HOTTIP, TUG1, ANRIL, PVT-1, AFAP1-AS1, ZFAS1 and CLMAT3 were associated with poor prognosis by promoting the proliferation and metastasis of CRC. Meanwhile, the decreased expressions of GAS5, LINC01296, LOC285194, RP11-462C24.1 and MEG3 were related to poor prognosis.

For OS, a significant degree of heterogeneity (*I*^2^ = 70%, *p*<0.001) was determined. Meta-regression analysis and subgroup analysis by sample size, analysis type, cut off values, and NOS score were also performed (Table 2). The cutoff value of mean indicated no statistical significance (HR = 0.296, 95% CI = 0.02–4.05). However, other factors including sample size, analysis type, NOS scores, and weight did not change the significant prognostic impact of high lncRNAs expression level on OS. Subgroup analysis and meta-regression analysis failed to reveal the source of heterogeneity (S1 Fig).

**Table 2:** Subgroup meta-analysis of pooled HRs for OS.

Categories	No. of studies	No. of patients	HR (95% CI) for OS	Meta-regression P-value	Heterogeneity	
					*I*^*2*^ (%)	*P*_*h*_
[1] OS	30	3361	2.08 (1.68–2.57)		70	<0.001
[2]No.of patients				0.715		
≥100	17	2500	2.16(1.62–2.88)		74	<0.001
<100	13	851	1.96 (1.46–2.63)		53	0.01
[3] Analysis type				0.863		
Multivariate	24	2862	2.09(1.64–2.68)		75	<0.001
Survival curves	6	951	1.76(1.37–2.26)		0	0.81
[4]Cut-off values				0.243		
Mean	3	298	0.29(0.02–4.05)		70	0.03
Median	10	1237	2.22(1.70–2.90)		54	0.02
Others	16	1943	2.17(1.60–2.95)		71	<0.001
[5] NOS score				0.853		
>7	11	1243	2.35(2.03–2.73)		68	0.0005
≤7	19	2298	2.06 (1.57–2.70)		69	<0.001
[6] Weight				0.726		
≥5%	5	615	1.95(1.33–2.86)		89	<0.001
<5%	24	2746	2.13(1.63–2.80)		61	<0.001

CRC: Colorectal Cancer; TNM: Tumor node metastasis; I^2^>50% with the random-effects model; I^2^<50% with the fixed-effects model.

**Fig 3:**
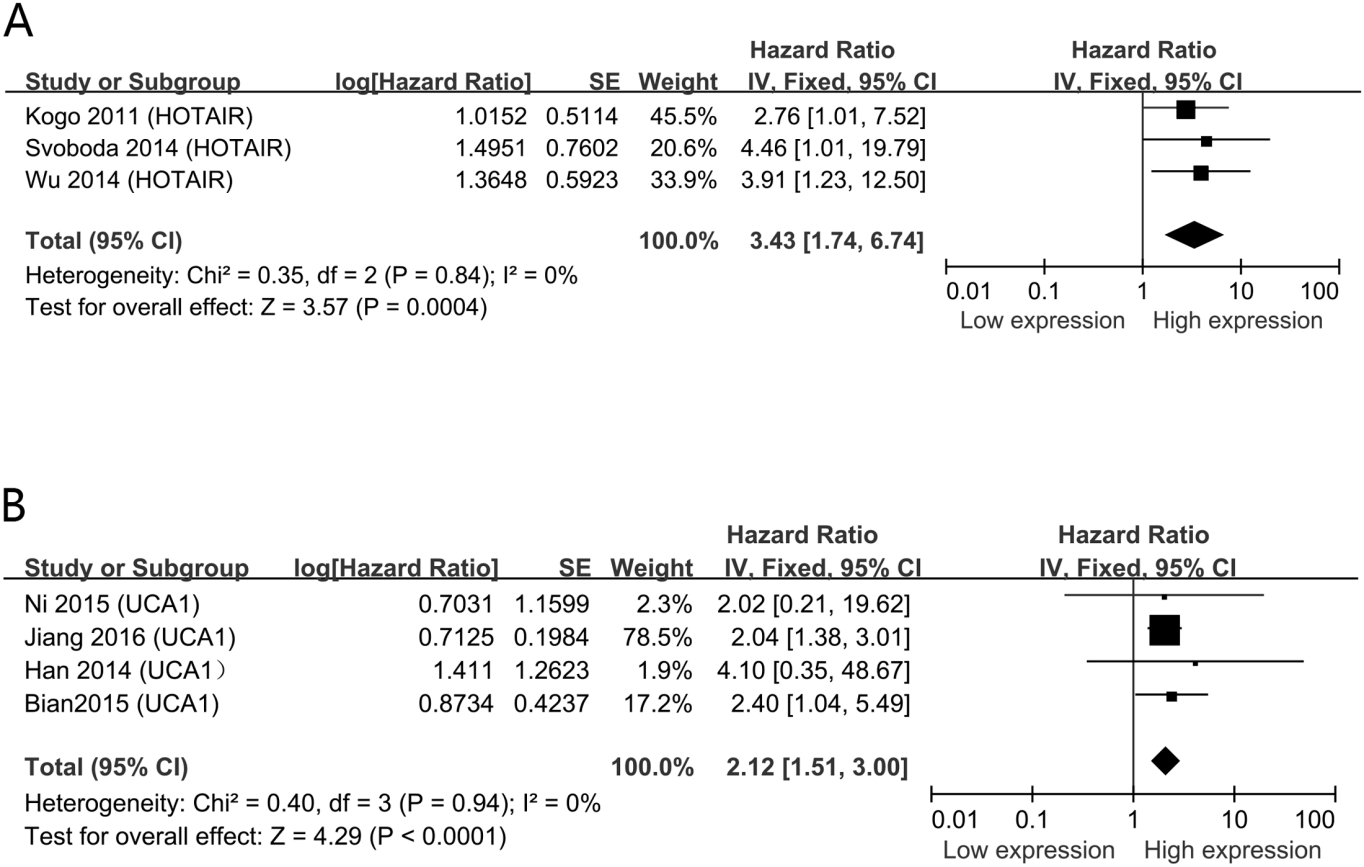
Forest plots of studies evaluating hazard ratios of up-regulated lncRNAs and the overall survival of CRC patients. A. HOTAIR; B. UCA1.

In the including studies, HOTAIR and UCA1 were investigated in three and four studies respectively. The other lncRNAs were performed in single study. We then conducted a meta-analysis on the relationship between HOTAIR/ UCA1 expression and OS of CRC patients. We found that high HOTAIR expression could predict short OS (HR = 3.43, 95% CI = 1.74–6.74, *P* = 0.0004, *I*^2^ = 0%) (Fig 3A). Besides, a poor prognosis in CRC was found in the upregulated levels of UCA1(HR = 2.12, 95% CI = 1.51–3.00, *P*<0.001, *I*^2^ = 0%) (Fig 3B).

**Fig 4:**
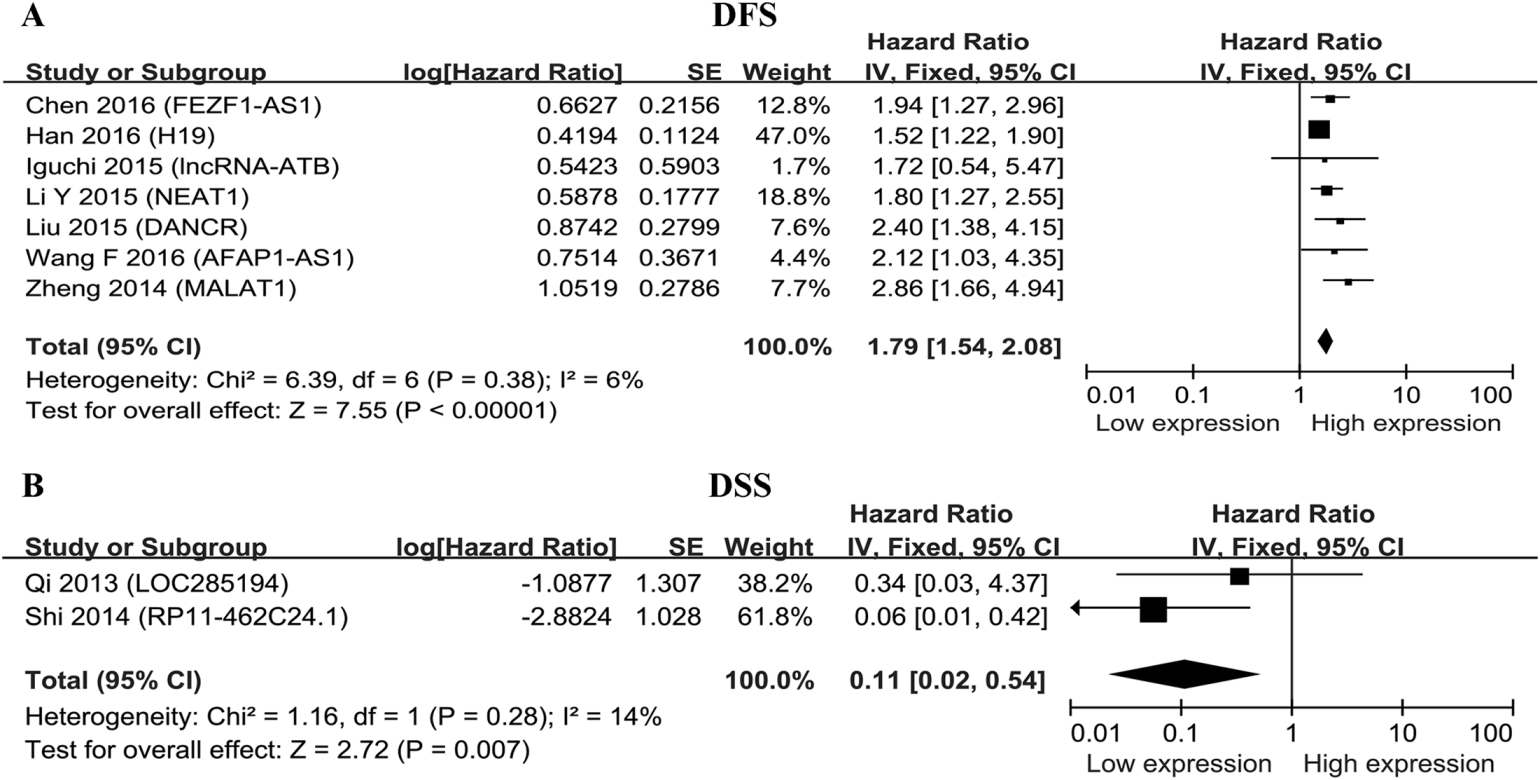
Meta-analysis of the pooled HRs of DFS and DSS for CRC patients. A.DFS; B. DSS.

The prognostic significance of lncRNAs in DFS was evaluated in seven studies[19, 25, 34, 36, 37, 42, 49] with 901 patients and that in DSS was examined in two studies [50, 51] with 167 patients (Table 1). There was only one study showed the association between lncRNAs expression level and RFS [24] and therefore was ruled out from the meta-analysis. The up-regulated expression of lncRNAs were significantly associated with DFS (HR = 1.79, 95% CI = 1.54–2.08, *P*<0.001, *I*^*2*^ = 6%) (Fig 4A), meanwhile, the down-regulated expression of lncRNAs was significantly correlated with DSS in CRC (HR = 0.01, 95% CI = 0.02–0.54, *P* = 0.007, *I*^*2*^ = 14%) (Fig 4B).

### Correlation of lncRNAs with clinicopathological characteristics

In order to determine whether the expression of lncRNA was related to the clinical pathological characteristics, the clinicopathological data was collected for the meta-analysis. As shown in Table 3, OR>1 implied that high levels of lncRNAs might be a risk factor in the features. In CRC, lncRNA transcription level was significantly associated with tumor differentiation (OR = 0.51, 95% CI = 0.34–0.77, P = 0.001, random-effect model), lymph node metastasis (OR = 1.63, 95% CI = 1.23–2.17, P = 0.0007, random-effect model), distant metastasis (OR = 2.06, 95% CI = 1.29–3.30, P = 0.002, random-effect model), TNM stage (OR = 0.44, 95% CI = 0.32–0.62, *P*<0.001, random-effect model), tumor invasion depth (OR = 0.48, 95% CI = 0.39–0.60, *P*<0.001, fixed-effect model), and tumor size (OR = 0.52, 95% CI = 0.31–0.88, *P* = 0.02, random-effect model). However, no significant correlation was found with gender (OR = 0.88, 95% CI = 0.76–1.02, *P* = 0.08, fixed-effect model) (S2 Fig).

**Table 3:** Association between high levels of lncRNAs and characteristics of patients with CRC.

Clinicopathological Parameters	Studies	Number of patients	Relative risk of higher lncRNAs OR (95% CI)	Significant Z	Test p-value	Heterogeneity I^2^ (%)	Test p-value	Model
Gender (Female vs. male)	29	3125	0.88(0.76–1.02)	1.74	0.08	0	0.66	Fixed effects
Tumor size (<5 vs ≥5)	7	672	0.52(0.31–0.88)	2.43	0.02	65	0.009	Random effects
Tumor differentiation (Moderate/well vs. poor)	16	1845	0.51(0.34–0.77)	3.24	0.001	70	<0.001	Random effects
Lymph node metastasis (Positive vs. negative)	24	2748	1.63(1.23–2.17)	3.38	0.0007	66	<0.001	Random effects
Distant metastasis (Positive vs. negative)	20	1998	2.06(1.29–3.30)	3.03	0.002	68	<0.001	Random effects
TNM stage (I–II vs. III–IV)	18	1770	0.44(0.32–0.62)	4.84	<0.001	62	0.0002	Random effects
Tumor invasion depth (T1-T2 vs. T3-T4)	17	1822	0.48(0.39–0.60)	6.66	<0.001	39	0.05	Fixed effects

Abbreviations: CRC: Colorectal Cancer; TNM: Tumor node metastasis; I^2^>50% with the random-effects model; I^2^<50% with the fixed-effects model.

### Sensitivity analyses

**Fig 5:**
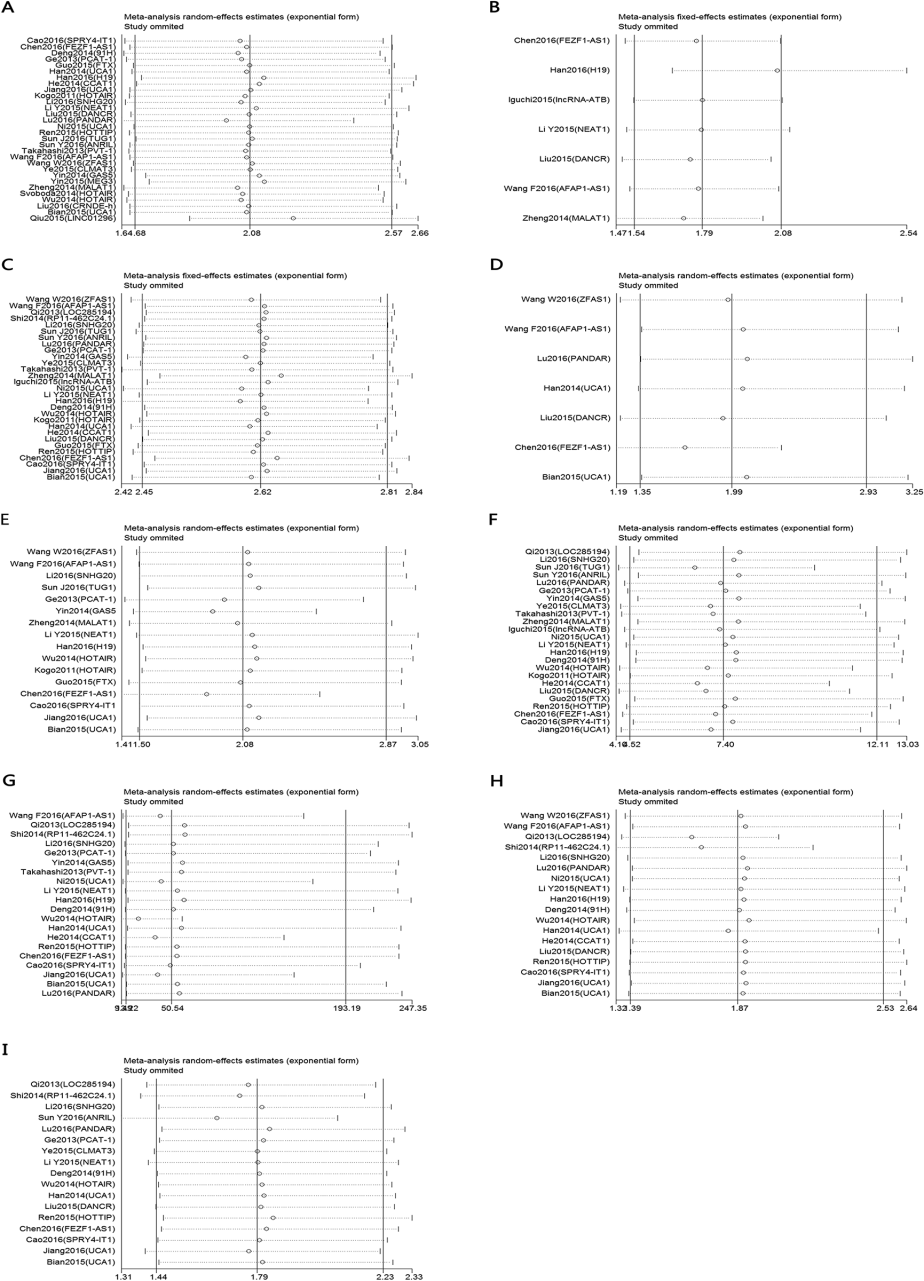
Sensitivity analyses of the studies. A. overall survival; B. disease-free survival; C. gender; D. tumor size (<5 vs ≥5); E. tumor differentiation; F. lymph node metastasis; G. distant metastasis; H. TNM stage; I. Tumor invasion depth.

To assess whether a single study might significantly affect the overall results, we performed a sensitivity analysis using Stata12.0 software. In the current study, removing any of the included studies had no significant influence on the estimated pooled results, which demonstrated that our analyses were relatively stable and credible (Fig 5). Because of the small number of studies, the sensitivity analysis was not analyzed for DSS.

### Publication bias

**Fig 6:**
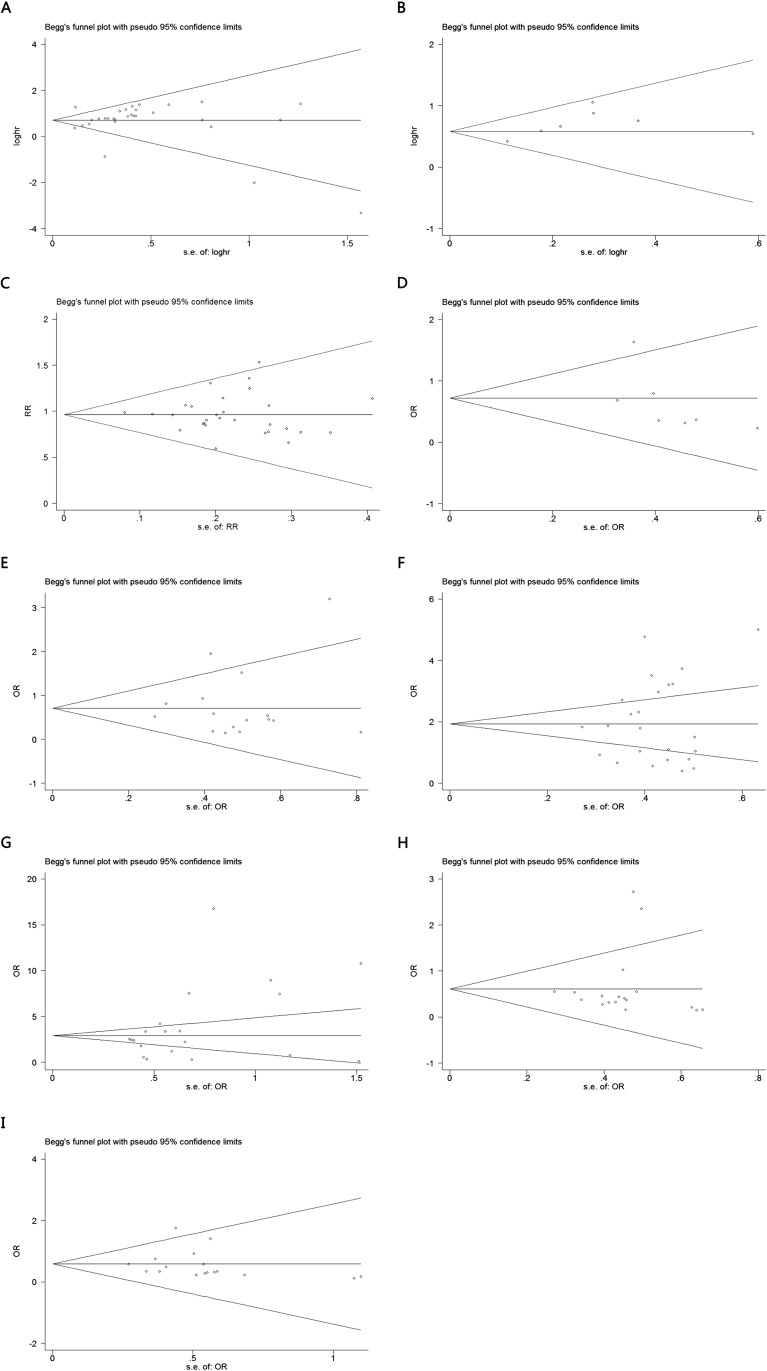
Begg’s test for publication bias. A. overall survival; B. disease- free survival C. gender; D. tumor size (<5 vs ≥5); E. tumor differentiation; F. lymph node metastasis; G. distant metastasis; H. TNM stage; I. Tumor invasion depth.

The publication bias of the present meta-analysis was evaluated by Bgger’s test. All groups had no bias, due to all the values of *P*>0.05(Fig 6). For DSS group, the publication bias was not analyzed because of the small number of studies.

## Discussion

Nowadays, CRC remains a major health problem and it is the third most common cause of cancer-related death wordwide[53–55]. Molecular Pathologic Epidemiology [56] is a relatively new, evolving field of epidemiology that is designed to clarify how various exposures affect initiation, transformation and progression of neoplasia[57]. It will continue to provide insights into carcinogenic process and help us to optimize prevention and treatment strategies[58]. Recently, a large number of studies demonstrated that the occurrence and progression of CRC was a multi-step process involving in lncRNAs dysregulation of multiple oncogenes and tumor suppressors. It is urgent to identify sensitive and specific biomarkers for early diagnosis and prognosis evaluation[35]. The up-regulation of FTX could serve as an important prognostic factor in CRC patients by promoting growth, migration, invasion and increasing colony formation in colorectal cancer cells[43]. Study from Cao *et al*. showed that up-regulated expression of SPRY4-IT1 dramatically shortened patients’ survival time by EMT pathway[45]. In the CRC, MEG3 might act as a tumor suppressor gene and contribute to tumorigenesis through inhibiting cancer cell proliferation[30]. In addition, some researchers pointed out that H19 was associated with a poor prognosis in colorectal cancer, which promoted tumor growth by recruiting and binding to eIF4A3[37]. In order to search for a prognostic potential target for CRC, we performed a meta-analysis to examine the relationship between lncRNAs and prognosis of patients with CRC.

Emerging evidence have demonstrated that the association between lncRNAs and CRC. In 2014, Yin *et al*. suggested that the downregulated GAS5 was related with a poor prognosis and could serve as a candidate prognostic biomarker for OS[29]. Meanwhile, the elevated expression of 91H was regarded as a novel prognosis indicator that contributed to predict tumor metastasis and poor survival of patients with CRC[18]. In our meta-analysis, we explored the prognostic role of lncRNAs in CRC. The results implied that high lncRNA transcription level exhibited a significant risk factor for OS, DFS and DSS. For OS, the test for heterogeneity of included studies was significant (*I*^2^ = 70, *P*<0.001). Although we employed subgroup analysis, meta-regression analysis and sensitivity analysis, all the methods failed to confirm the source of heterogeneity. For DSS, there were only two studies revealed its correlation with LncRNA expression, thus in the further, large-scale and more detailed studies may be needed to be recruited in the meta-analysis.

In our meta-analysis, HOTAIR was investigated in three studies and Urothelial carcinoma-associated 1 (UCA1) was detected in four studies. The increased expression of the two lncRNAs was associated with low survival rate of patients of CRC. UCA1 is an oncofoetal gene involved in embryonic development and carcinogenesis[35]. In 2015, Ni *et al*. illuminated that UCA1 could significantly enhance migration and invasion of CRC cells. Bian *et al*. found UCA1 promoted cell proliferation and chemoresistance in colorectal cancer by inhibiting miR-204-5p[48]. Furthermore, HOTAIR regulated expression of multiple genes in cooperation with PRC2 and was a novel molecule involved in the progression of CRC[40]. Therefore, it also was an independent prognostic factor for patients with CRC. Our results showed that the two lncRNAs had a significantly prognostic value in CRC.

In addition, we assessed the association of lncRNA transcription levels with the main clinicopathological features of CRC. Subgroup analysis indicated that lncRNA transcription was related to tumor size, tumor differentiation, lymph node metastasis, distant metastasis, TNM stage and tumor invasion depth. However, there was no correlation between lncRNAs expression and gender.

We have to admit that there are some limitations in our study. Firstly, most of the population in our studies were Chinese, so the conclusion of this study cannot be extended to all populations. Secondly, multiple lncRNAs were used to evaluate the prognosis of CRC, and there was a lack of specific CRC-related lncRNA for clinical evaluation. Thirdly, since there was only one study for each lncRNA in most cases, the prognostic value of each lncRNA may be overestimated. Furthermore, data of HR and 95% CIs were estimated from Kaplan–Meier survival curves in eight studies, which might be less accurate than that acquired directly from published statistics and might increase the potential bias.

Together, this meta-analysis for the first time evaluated the expressions of lncRNAs and clinical values of lncRNAs in CRC. The above analysis showed that lncRNAs were closely related with colorectal cancer, and it can be used as a promising indicator for prognostic of patients with CRC. However, larger-size and higher-quality studies are required to offer better insights into the prognostic value of lncRNAs patients with CRC.

## Supporting information

S1 Fig Forest plot of HRs for the association between lncRNAs expression levels and OS in cancer patients stratified by different subsets. A. sample size; B. analysis type; C. cut-off valus; D. Nos score; E. weight.
(TIF)

S2 Fig Forest plot for the association between lncRNAs expression levels with characteristics of patients with CRC. A. gender; B. tumor size (<5 vs ≥5); C. tumor differentiation; D. lymph node metastasis; E. distant metastasis; F. TNM stage; G. Tumor invasion depth.
(TIF)
