# Supplementary figures and images for: The prognostic value of abnormally expressed lncRNAs in colorectal cancer: A meta-analysis
Source: PLoS One. 2017 Jun 28;12(6):e0179670. doi: 10.1371/journal.pone.0179670 (PMC5489187; doi:10.1371/journal.pone.0179670)

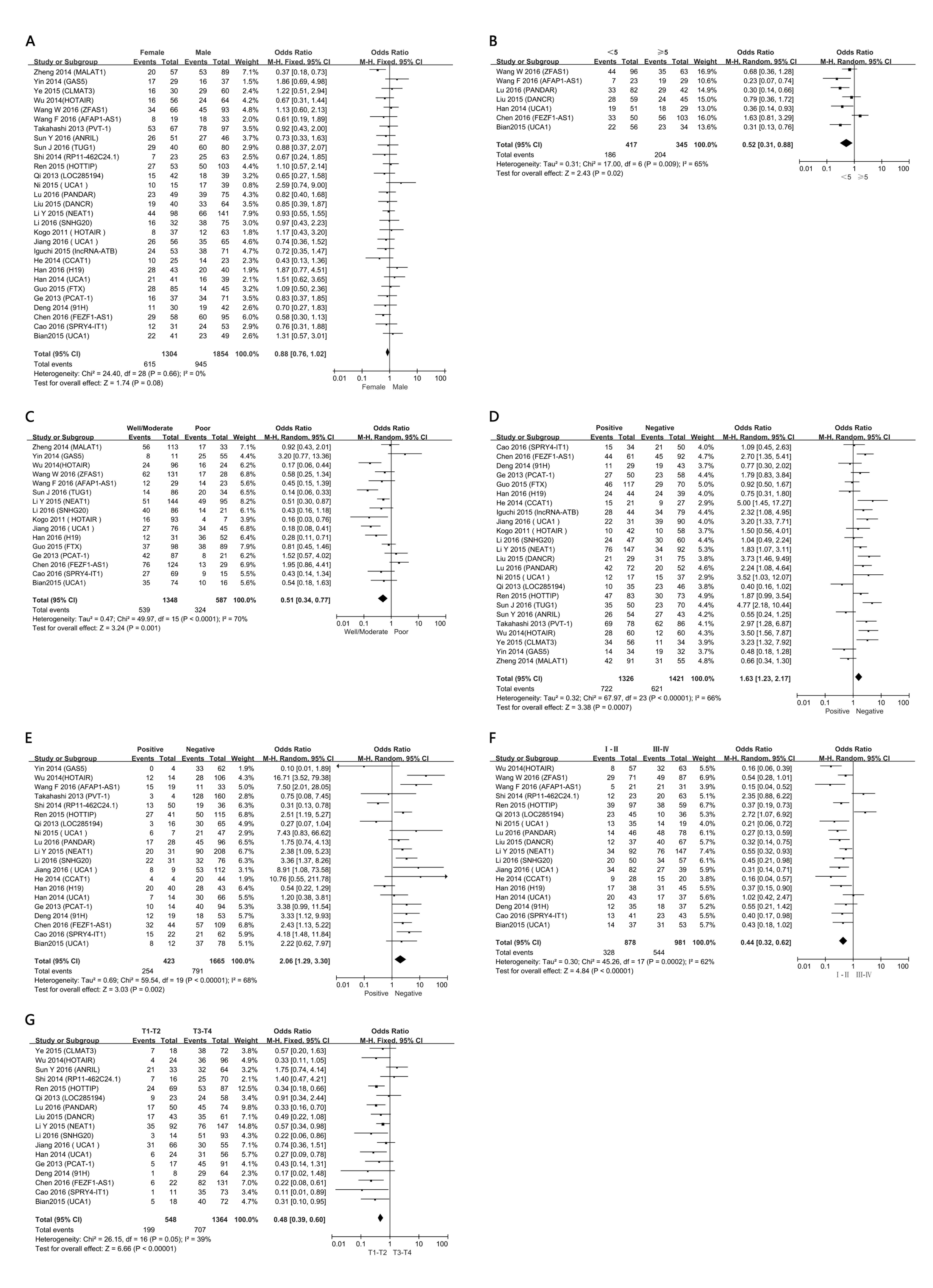

Supplement: S2 Fig — A. gender; B. tumor size (<5 vs ≥5); C. tumor differentiation; D. lymph node metastasis; E. distant metastasis; F. TNM stage; G. Tumor invasion depth. (TIF) [file pone.0179670.s002.tif]
